# Prenatal Exposure to Di-Ethyl Phthalate (DEP) Is Related to Increasing Neonatal IgE Levels and the Altering of the Immune Polarization of Helper-T Cells

**DOI:** 10.3390/ijerph18126364

**Published:** 2021-06-11

**Authors:** Chang-Ku Tsai, Hsin-Hsin Cheng, Te-Yao Hsu, Jiu-Yao Wang, Chih-Hsing Hung, Ching-Chang Tsai, Yun-Ju Lai, Yu-Ju Lin, Hsin-Chun Huang, Julie Y. H. Chan, You-Lin Tain, Chih-Cheng Chen, Ti-An Tsai, Hong-Ren Yu

**Affiliations:** 1 Department of Pediatrics, Kaohsiung Chang Gung Memorial Hospital, Kaohsiung 83301, Taiwan; wind518@cgmh.org.tw (C.-K.T.); hhuang@cgmh.org.tw (H.-C.H.); tainyl@hotmail.com (Y.-L.T.); charllysc@adm.cgmh.org.tw (C.-C.C.); tiantsai@cgmh.org.tw (T.-A.T.); 2 Graduate Institute of Clinical Medical Sciences, College of Medicine, Chang Gung University, Taoyuan City 33302, Taiwan; 3 Department of Obstetrics and Gynecology, Chang Gung Memorial Hospital-Kaohsiung Medical Centre, Kaohsiung 83301, Taiwan; chokovarous@cgmh.org.tw (H.-H.C.); tyhsu@adm.cgmh.org.tw (T.-Y.H.); aniki@cgmh.org.tw (C.-C.T.); lusionbear@hotmail.com (Y.-J.L.); lyu015@cgmh.org.tw (Y.-J.L.); 4 Department of Pediatrics, College of Medicine, National Cheng Kung University, Tainan City 704302, Taiwan; a122@mail.ncku.edu.tw; 5 Department of Pediatrics, Kaohsiung Medical University Hospital, Kaohsiung 80756, Taiwan; pedhung@gmail.com; 6 Institute for Translational Research in Biomedicine, Kaohsiung Chang Gung Memorial Hospital, Kaohsiung 83301, Taiwan; jchan@cgmh.org.tw

**Keywords:** cord blood, IgE, T-cell polarization, plasticizer, prenatal

## Abstract

**Introduction:** Phthalates are substances that are added to plastic products to increase their plasticity. These substances are released easily into the environment and can act as endocrine disruptors. Epidemiological studies in children have showed inconsistent findings regarding the relationship between prenatal or postnatal exposure to phthalates and the risk of allergic disease. Our hypothesis is that prenatal exposure to phthalates may contribute to the development of allergies in children. **Material and methods:** The objective of this study was to determine the associations between urinary phthalate metabolite concentrations in pregnant women, maternal atopic diathesis, maternal lifestyle, and cord blood IgE. Pregnant mothers and paired newborns (n = 101) were enrolled from an antenatal clinic. The epidemiologic data and the clinical information were collected using standard questionnaires and medical records. The maternal blood and urine samples were collected at 24–28 weeks gestation, and cord blood IgE, IL-12p70, IL-4, and IL-10 levels were determined from the newborns at birth. The link between phthalates and maternal IgE was also assessed. To investigate the effects of phthalates on neonatal immunity, cord blood mononuclear cells (MNCs) were used for cytokine induction in another in vitro experiment. **Results:** We found that maternal urine monoethyl phthalate (MEP) (a metabolite of di-ethyl phthalate (DEP)) concentrations are positively correlated with the cord blood IgE of the corresponding newborns. The cord blood IL-12p70 levels of mothers with higher maternal urine MEP groups (high DEP exposure) were lower than mothers with low DEP exposure. In vitro experiments demonstrated that DEP could enhance IL-4 production of cord blood MNCs rather than adult MNCs. **Conclusion:** Prenatal DEP exposure is related to neonatal IgE level and alternation of cytokines relevant to Th1/Th2 polarization. This suggests the existence of a link between prenatal exposure to specific plasticizers and the future development of allergies.

## 1. Introduction

Over the past several decades, there has been a consensus that the prevalence of atopic diseases is increasing rapidly in both Taiwan and other developing countries. In 1974, the prevalence of childhood asthma in Taipei, Taiwan, was 1.3%, and it subsequently increased to 5.0% in 1985, according to a report by Hsieh [1]. Between 1994 and 2002, in the same place, there was a consistent trend observed for asthma prevalence (14.4% in 2002) according to data collected using a standardized International Study of Asthma and Allergies in Childhood (ISAAC) questionnaire [2]. In a study in 2007, Wu et al. carried out an ISAAC-based survey of 24,999 first-grade elementary school students and found that the prevalence of diagnosed asthma, allergic rhinitis, and atopic eczema was 13.0%, 33.7%, and 29.8%, respectively, and that the increase in the prevalence of atopic eczema was statistically significant [3]. Therefore, the prevention and management of allergic diseases are important issues pertaining to the health-care system in Taiwan.

Plasticizers are a group of endocrine disrupting chemicals (EDCs) that are added during various manufacturing processes and seep easily into the environment; hundreds of million tons of EDCs are produced each year. Among the plasticizers, phthalates are the most common and are used extensively. Di-n-butyl phthalate (DnBP) and di-ethyl phthalate (DEP) are two common phthalates with measurable metabolites present in the urine [4]. DnBP is used mainly as a plasticizer and is also used as an additive in cosmetics, floor carpets, and in the coating of enteric medications [5]. DEP is commonly used in cosmetic products; however, it is also used in plastic packaging and medical devices [5]. DnBP has been shown to have adverse effects on the male reproductive system, and its use in children’s toys is prohibited in the European Union. A recent report has shown that the effects on the reproductive system are influenced by DEP as much as DnBP [6]. Similarly, Bisphenol A (BPA) is an xenoestrogen used in the manufacture of polycarbonate plastics and epoxy resins [7]. These materials are found in toys, drinking containers, dental sealants, water pipes, and even infant formulae. The extensive use of plastic materials may be related to an increased exposure to phthalate esters observed in Taiwan, thereby increasing the risk of the adverse effects of phthalates, particularly in pregnant women and children [8].

Many studies have been conducted to address the safety aspects related to phthalates on fetal development and postnatal health. However, the importance of the clinical relevance of real-life exposure and the identification of molecular targets that can explain the interactions largely remains to be investigated. vom Saal found that the conventional risk-assessment strategies used for most chemicals cannot be used to assess the toxicity of endocrine-active compounds, because such compounds often display nonmonotonic dose responses [9]. Therefore, not all epidemiologic studies have revealed consistent results with endocrine-active compounds, which may be related to the differences in the exposure periods and the exposure doses to plasticizers. In real life, the exposure to environmental conditions is continuous from the fetus stage to the postnatal period. A recent study has shown that urinary mono (2-ethylhexyl) phthalate (MEHP) in pregnant women and 5-year-old children was significantly associated with increased IgE in children suffering from allergies at the age of 8 years [10]. Additionally, it is observed that in children, exposure to phthalates at different ages also leads to different outcomes of phthalate exposure [11,12]. However, the effects of exposure to other phthalates during the fetal stage on the development of atopy later in life remain unclear. Therefore, the mechanism by which fetal phthalate exposure leads to atopy development needs to be further explored. Here, we conducted two parallel studies, one based on a birth cohort study, and the other based on an in vitro model. In the cohort study, maternal atopic status and lifestyle, maternal phthalates and BPA exposure, maternal IgE, and the neonatal immune health effects (IgE/cytokine) from the mothers were analyzed. The in vitro study was intended to provide direct evidence for the susceptibility of neonates to phthalate exposure.

## 2. Materials and Methods

### 2.1. Study Population

The participants in this pregnancy–birth cohort study was selected between August 2016 and July 2018 at an antenatal clinic in Kaohsiung Chang Gung Memorial Hospital, a medical center located in southern Taiwan. This protocol was approved by the Institutional Review Board of Chang Gung Memorial Hospital, Kaohsiung Medical Center (IRB: 104-9574A3). All the participants gave written informed consent prior to enrolment. We recruited pregnant Taiwanese women without clinical complications (eclampsia or preeclampsia) at 24–28 weeks gestation, who delivered their babies at the Kaohsiung Chang Gung Memorial Hospital. Maternal venous blood (10 mL) and spot urine (10 mL) were collected at 24–28 weeks of gestation during regular antenatal visits. The samples were immediately transferred to the laboratory and stored at −20 °C before analysis. In some cases, human umbilical cord blood was obtained at the time of delivery from neonates born by uncomplicated deliveries as previous reported [13].

### 2.2. Questionnaire

We conducted face-to-face interviews with the participants to gather maternal demographic and socioeconomic characteristics (such as maternal age, ethnicity, and education), disease history and lifestyle during the pregnancy (alcohol consumption and smoking, home and external environments, such as humidity, etc.) by a standardized questionnaire in the second trimester.

### 2.3. Measurement of Total IgE in Maternal Plasma and Cord Blood Plasma

The total IgE levels from the blood of mothers and the cord blood of their newborns were measured using the ImmunoCAP (Phadia AB, Uppsala, Sweden) assay with a detection limit of 0.005 kU/L. The IgA level was detected to ensure the cord blood samples were free from contamination by the maternal blood (less than 10 μg/mL) [14].

### 2.4. Measurement of Cytokines of Maternal Plasma and Cord Blood Plasma

The human umbilical cord blood was collected from healthy mothers at the time of their elective cesarean section or normal spontaneous delivery after obtaining their informed consent. Plasma cytokines IL-4 (eBioscience, Vienna, Austria), IL-10, and IL-12p70 (all from R&D systems Inc., Minneapolis, MN, USA) in the maternal and cord blood samples were determined by enzyme-linked immunosorbent assay (ELISA).

### 2.5. Measurement of Phthalate Metabolites and BPA in Urine

Urine samples were used for the measurement of phthalate metabolites, including monobenzyl phthalate (MBzP), monoethyl phthalate (MEP), mono-n-butyl phthalate (MnBP), mono (2-ethylhexyl) phthalate (MEHP), and Bisphenol A (BPA) using liquid chromatography and electrospray ionization tandem mass spectrometry. The metabolites were quantified by an internal isotope standard curve method employing a slightly modified method of Blount et al. [15]. Briefly, each urine sample was fortified with 50 μL of internal standard (200 ng/mL, mixed with phthalate metabolites-13C12) spiking solution, 1 mL of 2 M ammonium acetate buffer solution (1.54 g of ammonium acetate/10 mL HPLC-grade water), and 20 μL of β-glucuronidase. The samples were incubated for 1 h at 37 °C, and then extracted twice with 4 mL of ethyl acetate. Next, the samples were gently shaken a few times, and the organic layer was separated from the non-polar fat layer by centrifugation at 4000 rpm for 15 min. Chromatographic separation was performed on a Shim-pack GIST C18 column (2.1 mm × 100 mm, 2 μm; Shimadzu, Japan). The analysis of the target compound was performed using a Shimadzu 8050 Triple Quad liquid chromatograph mass spectrometer equipped with a Shimadzu LC-30 series HPLC system. The detection limit of MEP and BPA was 1 μg/μL. The detection limit for MnBP, MBzp, and MEHP was 0.8 μg/μL.

### 2.6. Induction of Cytokine Release by MNCS

For the in vitro study, atopic/allergic mothers were excluded to avoid underlying interference. These cord blood leukocytes and red blood cells were separated from whole blood using 4.5% (*w*/*v*) dextran. MNCs were further separated by density gradient centrifugation using Ficoll-Paque™ at a ratio of 2:1 as previously described [13,16]. For cytokine induction, cord blood MNCs were suspended to 2 × 10^6^ cells/mL in a 24-well plate in an RPMI-1640 medium (supplemented with 10% heat-inactivated fetal bovine serum, 1 mm glutamine, 100 IU/mL penicillin and 100 μg/mL streptomycin) and pre-incubated with DEP (AccuStandard, New Haven, CT, USA) at concentrations of 0, 10^−9^, 10^−8^, 10^−7^, 10^−6^ mM for 30 min. The cells were then stimulated with 10 μg/mL of phytohemagglutinin (PHA) (Sigma-Aldrich, St. Louis, MO, USA). After 72 h, cell-free culture supernatants were collected and assayed for cytokine production, such as IL-4 (eBioscience, Vienna, Austria) and IFN-γ (R&D systems Inc., Minneapolis, MN, USA) by ELISA.

### 2.7. Statistical Analysis

Means ± standard deviation (SD) or numbers (frequency) were tabulated to describe the demographic characteristics and the urinary metabolites where appropriate. Continuous variables were expressed as means ± standard error. The Mann–Whitney U test was used to determine the continuous variables between the groups. Spearman correlation coefficients were used to assess the correlation between the five urinary phthalate metabolites and the total IgE levels in the plasma samples of pregnant women and the cord blood of their offspring. The data were analyzed using the IBM SPSS Statistics version 22. A *p* value of <0.05 indicated statistical significance.

## 3. Results

### 3.1. Demographic Characteristics of Participants

In total, 126 pregnant Taiwanese women were assessed from August 2016 to July 2018. After excluding participants from whom cord blood samples of their newborns were not obtained, 101 mothers paired with their newborns were enrolled. The mean age (±SD) of the participant mothers was 34.12 (±3.99) years with a range of 23–42 years (Table 1), and their average BMI at delivery was 27.08 ± 4.42 kg/m^2^. Among them, seven mothers (6.9%) had asthma, 35 (34.7%) had allergic rhinitis, and 16 (15.8%) had atopic dermatitis before pregnancy. The gestational age of the newborns was 38.04 ± 2.42 weeks with an average birth height and birth weight of 48.34 ± 3.04 cm and 2932.66 ± 555.48 g, respectively (Table 1).

### 3.2. Urinary Phthalate Metabolites

After correction for urinary creatinine, the median levels of the five urinary phthalate metabolites (μg/g creatinine) were 9.80 for MEP, 6.20 for MnBP, 0.25 for MBzP, 1.70 for MEHP, and 1.58 for BPA (Table 2).

### 3.3. Plasma Total IgE Levels

The median level of the total IgE in the maternal plasma was 38.20 KU/L, whereas it was 0.20 KU/L in the cord blood plasma.

### 3.4. Correlation of Maternal Characteristics with Maternal Phthalate Metabolites, Maternal IgE, and Cord Blood IgE

We found that older mothers (>35 years old) showed significantly higher concentrations of BPA (Table 3). Pregnant women who had a history of allergic rhinitis tended to exhibit lower mean concentrations of MnBP and MBzP. The IgE levels of mothers who had a history of asthma or allergic rhinitis were significantly higher (*p* < 0.05) than those who did not. Moreover, pregnant women who often consumed shellfish showed significantly higher concentrations of MnBP (*p* < 0.05). In addition, higher levels of IgE in the plasma samples were observed in the pregnant women who often consumed fish. We did not observe statistically significant differences between IgE levels and maternal education, the presence of furry pets at home during pregnancy, and the exposure of the family to smoking. In addition, we observed that maternal lifestyle did not have a significant impact on the cord blood IgE levels of newborns. Detailed information on the distribution of prevalence is presented in Table 3.

**Figure 1:**
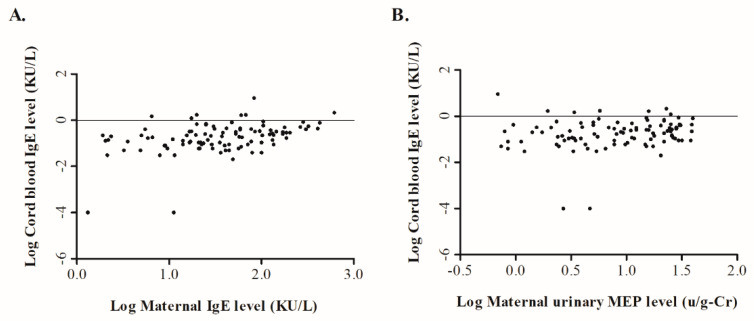
Correlation of cord blood IgE levels with the corresponding maternal IgE and maternal urine monoethyl phthalate (MEP) levels. One hundred and one pairs of maternal plasma and urine samples collected at 24 to 28 weeks of gestation and cord blood samples of their neonates at birth were collected. The correlation of (**A**) cord blood IgE with corresponding maternal IgE and (**B**) maternal urine MEP are shown.

The total maternal IgE level was significantly and positively correlated with the IgE level of the corresponding cord blood levels of their offspring (*r* = 0.393, *p* < 0.001) (Figure 1A). No association was found between urinary phthalate metabolites and the total maternal IgE. However, a significant positive correlation among the five urinary phthalate metabolites was observed. Table 4 shows the correlation between urinary phthalate metabolites in pregnant mothers and the IgE levels in maternal and cord blood plasmas. We found that urinary MEP in pregnant mothers was the only phthalate metabolite that was significantly correlated with the level of cord blood IgE (*r* = 0.226, *p* = 0.023) (Figure 1B).

### 3.5. Correlation of Maternal Phthalate Metabolites, Maternal and Cord Blood Cytokines

Table 5 shows the correlation between the urinary phthalate metabolites in pregnant mothers and the plasma cytokines level in the maternal and the cord blood plasmas. The IL-4 level was almost undetectable even with high sensitivity ELISA (assay range 0.3–16 pg/mL). There was no association between any of the five urinary phthalate metabolites and the maternal plasma IL-10 level, or the IL12p70 level. In contrast, we found that the urinary MEP in pregnant mothers was positively correlated with the level of the cord blood IL-10 level (*r* = 0.221, *p* = 0.032), and was negatively corelated with the cord blood IL-12p70 level (*r* = −0.309, *p* = 0.018).

**Figure 2:**
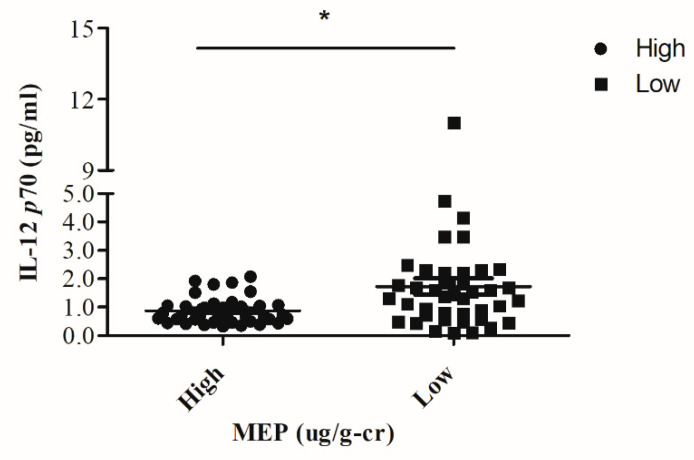
The study cases were divided into two groups according to the median value (9.2 μg/g-cr) of the maternal urine MEP level. The high maternal urine MEP group showed a lower IL-12p70 level of paired cord blood plasma as compared with the low maternal urine MEP group (0.92 ± 0.01 pg/mL vs. 1.73 ± 0.01 pg/mL, *p* = 0.014). The cord blood plasma IL-12p70 levels were determined by ELISA. The * *p* value was determined with the Mann–Whitney U test.

After dividing these mothers into high and low DEP exposure groups according to the median value of the urine MEP (9.2 μg/g-cr), we found that the high maternal urine MEP group had a lower IL-12p70 plasma level in the paired cord blood plasma compared to the low maternal urine MEP group (0.92 ± 0.01 pg/mL vs. 1.73 ± 0.01 pg/mL, *p* = 0.014) (Figure 2).

### 3.6. Effects of DEP on Th1/Th2-Related Cytokine Production by Cord Blood MNCs

**Figure 3:**
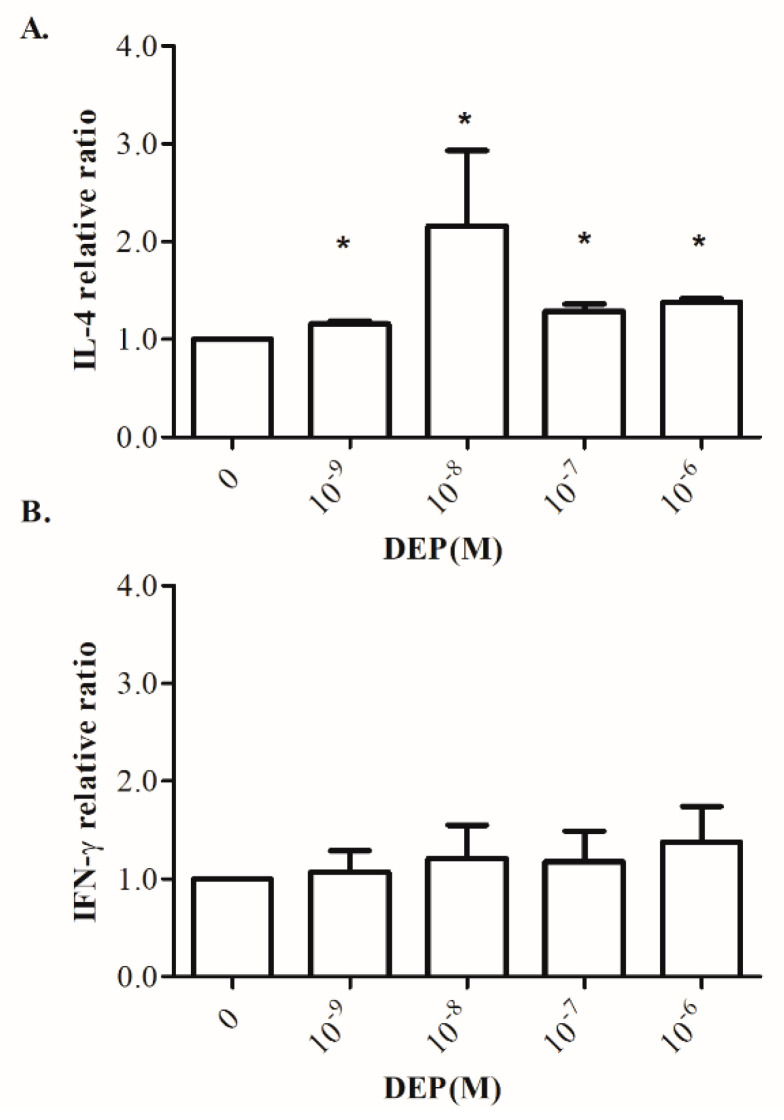
Effects of di-ethyl phthalate (DEP) supplementation on interleukin (IL)-4 and interferon gamma (IFN-γ) production by neonatal mononuclear cells (MNCs). MNCs isolated from cord blood were suspended at a concentration of 2 × 106/mL in 24-well plates and treated with 10 µg of phytohemagglutinin and the indicated concentrations of DEP for 72 h. Culture supernatants were collected and the levels of (**A**) IL-4 and (**B**) IFN-γ were determined by ELISA. (*n* = 6–8). * *p* < 0.05 compared to the control without DEP by ANOVA.

Since MEP is a metabolite of DEP, DEP was used for a further in vitro study. To clarify the modulatory effects of DEP on the Th1/Th2 immune polarization, we determined the levels of IL-4 and IFN-γ production by cord blood MNCs, which were exposed to different DEP levels upon PHA stimulation. As shown in Figure 3A, exogenous DEP enhanced IL-4 production by cord blood MNCs upon PHA stimulation, which attained the highest level at the concentration of 10^−8^ M, whereas exogenous DEP had no effect on IFN-γ production (Figure 3B).

## 4. Discussion

In this study, we investigated the effects of prenatal phthalates and BPA exposure on the IgE level at birth and the correlation between maternal characteristics and urinary phthalate metabolite concentrations. We found that pregnant women who were older than 35 years had higher urinary BPA levels than those aged less than or equal to 35 years. It was observed that the mothers who often consumed shell seafood had higher urinary MnBP levels.

**Figure 4:**
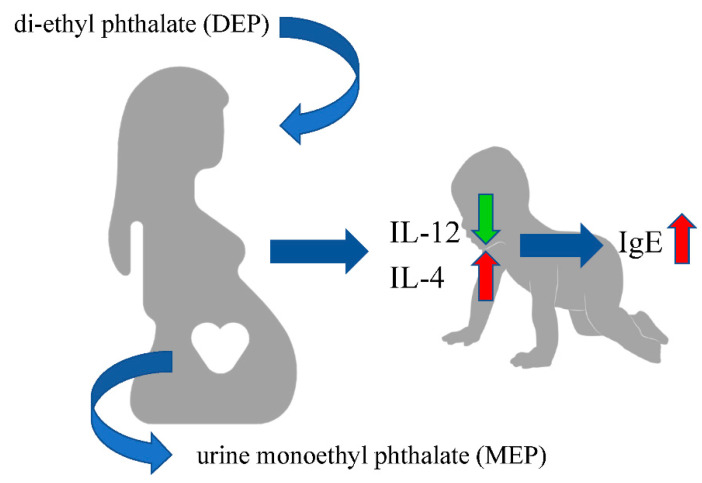
The possible link between prenatal DEP exposure and alternation of Th1/Th2 polarization related cytokines and neonatal IgE level.The risk of developing allergic disease is likely to be multifactorial, depending on individual genetic susceptibility and environmental exposure. Many risk factors related to childhood allergic diseases have been demonstrated, which include genetic atopic predisposition, early childhood allergen exposure and sensitization, occurrence of viral respiratory infections in young children, maternal smoking during pregnancy, poor dietary factors, lack of breast-feeding, childhood obesity, having a certain immunologic (Th2-prone) predisposition, air pollution, and frequent immunizations in childhood [17]. Our study demonstrated a positive association between IgE in the maternal plasma and cord blood IgE. This result is consistent with the studies of Nabavi et al. and Amici et al., which suggest that the transfer of cytokines and chemokines through the placenta and the gaining of access to the gut-associated lymphoid tissue can lead to the overproduction of IgE [18,19]. However, in our study, we found that the maternal urine MEP level only positively correlated with cord blood IL-12p70 levels but not with the level of IL-12p70 in the maternal plasma. The positive correlation of maternal plasma IgE with the cord blood IgE can also be partially explained by genetic atopic predisposition, because mothers with atopy often have higher IgE levels.

Our result revealed that there are positive correlations among several different phthalate metabolites. The maternal urinary MEP concentrations were positively correlated with cord blood IgE levels and were negatively correlated with cord blood IL-12p70 levels. After performing an in vitro experiment, we found that the indicated concentration of DEP, a precursor of MEP, could enhance the production of IL-4 by the cord blood MNCs. Taken together, these results indicated that modulation of Th1/Th2 related cytokines production may be a possible mechanism that leads to an increase in the cord blood IgE with prenatal DEP exposure (Figure 4).

The Developmental Origins of Health and Disease (DOHaD) theory hypothesizes that a chronic disease risk in neonates is influenced by environmental exposure in utero that alters fetal programming [20]. This hypothesis has been proven in several instances; one such example is environmental contaminant exposure in utero, which may alter the development of the immune system of newborns, thereby predisposing the newborns to have an allergic phenotype [21]. The potential role of phthalate exposure in childhood allergy development is supported by an experimental study, which indicated that these chemicals exhibit adjuvant-like properties [22]. However, epidemiological evidence regarding the effects of prenatal phthalate exposure are inconsistent. Gascon et al. reported that maternal urinary ΣDEHP metabolites in pregnancy were associated with an increased risk of wheezing and bronchitis during childhood [23]. In contrast, no significant association was noted between the maternal urinary MEP concentrations and asthma-like symptoms in children aged 5–11 years in a birth cohort study in the USA [24]. Furthermore, another birth cohort study from Taiwan showed that there was no significant correlation between the urine phthalate metabolite concentrations (MEP, MBP, MBzP, MEHP) at pregnancy and atopic dermatitis in the newborns [25]. These inconsistent results may be due to different study designs and genetic backgrounds. Hence, universal standards for disease risk evaluation are necessary for performing studies on prenatal phthalate exposure. The cord blood IgE is of fetal origin, and maternal IgE cannot be transferred to a child in the uterus [26]. To avoid the interference of postnatal environmental exposure while investigating the risk of prenatal phthalate exposure towards predisposition to allergies, we determined the cord blood IgE level using an early atopy marker. Another reason to study cord blood IgE was that it is relatively objective, comparable, and easily available.

In general, the phthalate metabolites have relatively short half-lives (6–12 h) [27]. A previous study that was designed to explore the reproducibility of phthalate levels in urine samples demonstrated a high degree of reliability in urinary phthalate levels from one day to the next, suggesting that the daily phthalate exposure patterns were relatively stable [28]. Hauser, R. et al. also showed that a single spot urine sample could accurately classify phthalate exposure over the previous 3 months [29]. For the stability of urinary phthalate metabolites during pregnancy, although there were some significant trends in the phthalate levels across gestation, most levels were relatively stable within the individual over time (especially for DEHP, MBzP, MBP, and MEP) [30,31]. However, other studies have suggested that a single urine measurement is not representative of long-term exposure [32]. Due to reasons related to research funding, our study was conducted to determine the exposure to phthalates during the second trimester. Further study with repeated measures for phthalate metabolites may clarify the effects of long-term exposure.

It is well known that cord blood IgE is a risk marker for allergic sensitization and asthma [33,34]. However, the association between the levels of maternal phthalates and cord blood IgE is still not clear. Three birth cohort studies in Japan, Taiwan, and Canada examined the association between prenatal phthalate exposure and cord blood IgE levels [25,35,36]. The Japanese birth cohort reported that cord blood IgE levels were negatively associated with maternal MEHP levels [35]. The Canadian study reported an inverse and non-linear association between maternal urinary mono-carboxypropyl phthalate (MCCP) levels and the total cord blood IgE level [36]. In contrast, the Taiwanese birth cohort study reported no statistically significant association between maternal phthalate metabolites and cord blood IgE [25].

The expansion of allergen-specific Th2 cells results in the production of IL-4, which induces immunoglobulin class switching to IgE and the clonal expansion of naive and IgE-producing memory B-cell populations. Allergen-specific IgE can bind to high-affinity receptors of IgE (FceRI) present on mast cells, basophils, and many other cells. Further allergen exposure induces cross-linking of the receptor-bound IgE, leading to cell degranulation and the release of proinflammatory molecules, such as histamine, which are responsible for the signs and symptoms of the immediate phase of the allergic reactions [37,38]. In our in vitro study, we found that exogenous DEP can induce IL-4 production by cord blood MNCs upon adequate activation; the highest level of IL-4 is attained at a concentration of 10^−8^ M. However, exogenous DEP had no effect on IFN-γ production. Some in vivo and in vitro studies have demonstrated that several phthalates have adjuvant effects on Th2 differentiation, Th2 cytokine induction, and the Th2-promoted antigen-specific production of IgE in mice [39]. However, reports related to the immune modulatory effect of phthalates on human cells are quite limited. Hansen et al. have shown that the secretion of IL-4 and IFN-γ is significantly impaired by adult MNCs treated with either DEP or DnBP [40]. The inconsistency observed in this study and our cord blood MNCs data suggests that the immune system of neonates is more susceptible to phthalates than that of adults. IL-12 is a Th1 promoting cytokine that is produced by activated antigen-presenting cells, such as dendritic cells and macrophages [41]. IL-12 can reduce the IL-4-mediated suppression of IFN-γ [42]. The maternal urine MEP level is negatively related to cord blood IL-12p70. Thus, DEP may induce allergy-related inflammation, which occurs through the suppression of Th1 and the induction of Th2 polarization.

Significantly positive correlations between the various urinary phthalate metabolites were observed in this study, and similar findings have been noted in other reports [32,43]. It is known that phthalates are ubiquitously present in the environment, and humans are exposed to a mixture of such chemicals rather than just a single phthalate [44]. This finding suggests that a plasticized material contains more than one kind of phthalate; thus, people who rely on plastic products in their daily life may have an increased exposure to a mixture of phthalates.

Food ingestion has been reported to be a major source of phthalate exposure [45]. Several studies have indicated positive associations between the consumption of seafood and phthalate metabolite concentration levels [46,47]. Our study agrees with these reports; the large amount of shell seafood consumption shown in this study was significantly associated with higher levels of MnBP. Phthalates may be present in wastewater that is discharged into streams, rivers, lakes, and oceans via untreated sewage, and may also be present in disposed garbage. Therefore, seafood is likely exposed to widespread phthalates in the aquatic environment [45,48].

The present study has several limitations. One limitation is the relatively small sample size that may have resulted in insufficient statistical power to conduct further stratification analyses. However, despite the small sample size, significant associations between phthalate metabolites and the total cord blood IgE level were observed. Another limitation is the collection of one-time urine samples only for the estimation of phthalate metabolite levels. Consequently, random variations of exposure to phthalates may have been underestimated in our findings.

## 5. Conclusions

In conclusion, our study data showed pregnant mother urine MEP level, a metabolite of DEP, is related to the neonatal IgE level and the alternation of Th1/Th2 polarization related cytokines. This study provides a possible mechanism to explain the link between the prenatal exposure to specific plasticizers and the development of allergies.

## Figures and Tables

**Table 1:** Demographic characteristics of the participant mothers and their newborns.

Variable	Subjects (*n* = 101)
	Mean ± SD or *n* (%)
Mother	
Age at delivery (years)	34.12 ± 3.99
BMI at delivery (Kg/m^2^)	27.08 ± 4.42
History of atopic disease	
Asthma	7 (6.9)
Allergic rhinitis	35 (34.7)
Atopic dermatitis	16 (15.8)
Newborn	
Gestation age (weeks)	38.04 ± 2.42
Birth height (cm)	48.34 ± 3.04
Birth weight (g)	2932.66 ± 555.48
Birth head circumference (cm)	33.16 ± 1.78

Abbreviation: SD = standard deviation; BMI = body mass index.

**Table 2:** Distribution of maternal urinary phthalate metabolites, and the maternal and corresponding newborn cord blood IgE levels.

Variables				Percentiles		
	*n*	Min	25th	50th	75th	Max
MEP (μg/g-creatinine)	101	0.69	3.38	9.80	22.00	39.40
MnBP (μg/g-creatinine)	101	0.01	3.59	6.20	13.75	67.00
MBzP (μg/g-creatinine)	101	0.02	0.14	0.25	0.46	1.36
MEHP (μg/g-creatinine)	101	0.01	0.65	1.70	3.04	19.10
BPA (μg/g-creatinine)	101	0.001	0.86	1.58	3.07	51.90
Maternal IgE (KU/L)	101	1.00	16.65	38.20	97.30	616.00
Cord blood IgE (KU/L)	101	0.0001	0.09	0.20	0.41	9.00

Abbreviations: Max = Maximum; Min = Minimum; MEP = Mono-ethyl phthalate; MnBP = Mono-n-butyl phthalate; MBzP = Mono-benzyl phthalate; MEHP = Mono (2-ethylhexyl) phthalate; BPA = Bisphenol A; IgE = Immunoglobulin E.

**Table 3:** Correlations among maternal phthalate metabolites, maternal IgE, maternal characteristics, and cord blood IgE.

Characteristics	%	MEP	MnBP	MBzP	MEHP	BPA	Maternal IgE	Cord Blood IgE
Age at delivery (years)								
≤35	59.4	12.24 ± 1.27	9.57 ± 1.34	0.32 ± 0.03	2.34 ± 0.34	1.94 ± 0.28 *	80.24 ± 13.75	0.52 ± 0.16
>35	40.6	14.22 ± 1.89	9.77 ± 1.41	0.31 ± 0.04	2.96 ± 0.64	4.22 ± 1.35	65.66 ± 14.19	0.25 ± 0.05
Maternal education								
<high school	9.9	12.9 ± 2.95	15.92 ± 6.09	0.37 ± 0.06	2.43 ± 0.85	2.18 ± 0.37	117.94 ± 44.48	0.46 ± 0.14
≥high school	90.1	13.06 ± 1.15	8.96 ± 0.85	0.31 ± 0.03	2.61 ± 0.35	2.94 ± 0.64	69.53 ± 9.92	0.40 ± 0.10
Maternal history of allergies								
Asthma								
Yes	6.9	13.45 ± 4.49	14.27 ± 8.98	0.36 ± 0.18	2.29 ± 1.23	1.92 ± 0.40	178.23 ± 52.42 ^†^	0.50 ± 0.12
No	93.1	13.01 ± 1.11	9.31 ± 0.83	0.31 ± 0.02	2.61 ± 0.34	2.94 ± 0.62	66.58 ± 9.62	0.40 ± 0.10
Allergic rhinitis								
Yes	34.7	12.84 ± 2.02	6.09 ± 0.81 ^†^	0.27 ± 0.05 *	2.57 ± 0.65	1.85 ± 0.24	113.06 ± 22.95 ^†^	0.57 ± 0.26
No	65.3	13.15 ± 1.26	11.54 ± 1.38	0.34 ± 0.03	2.60 ± 0.37	3.41 ± 0.87	53.78 ± 8.33	0.33 ± 0.05
Atopic dermatitis								
Yes	15.8	14.50 ± 2.77	8.36 ± 1.75	0.45 ± 0.09	3.00 ± 0.86	1.84 ± 0.35	97.92 ± 36.46	0.48 ± 0.15
No	84.2	12.77 ± 1.17	9.89 ± 1.11	0.29 ± 0.02	2.52 ± 0.36	3.06 ± 0.69	69.88 ± 9.72	0.40 ± 0.11
Maternal dietary intake								
Milk								
Seldom	24.8	10.03 ± 1.73	8.87 ± 1.67	0.30 ± 0.04	2.05 ± 0.37	3.72 ± 2.03	70.64 ± 18.03	0.34 ± 0.08
Often	75.2	14.03 ± 1.30	9.91 ± 1.18	0.32 ± 0.03	2.77 ± 0.42	2.59 ± 0.40	75.53 ± 11.92	0.43 ± 0.12
Egg								
Seldom	12.9	8.79 ± 1.87	9.47 ± 1.55	0.30 ± 0.04	1.93 ± 0.42	1.62 ± 0.37	87.77 ± 33.23	0.29 ± 0.11
Often	87.1	13.67 ± 1.19	9.68 ± 1.10	0.32 ± 0.03	2.69 ± 0.37	3.05 ± 0.66	72.33 ± 10.41	0.43 ± 0.11
Nut								
Seldom	77.2	12.28 ± 1.24	8.85 ± 0.86	0.30 ± 0.03	2.56 ± 0.39	2.34 ± 0.36	79.12 ± 12.15	0.41 ± 0.12
Often	22.8	15.60 ± 2.13	12.39 ± 3.13	0.36 ± 0.06	2.69 ± 0.59	4.64 ± 2.23	58.05 ± 14.81	0.42 ± 0.09
Shell seafood								
Seldom	91.1	12.46 ± 1.10	8.80 ± 0.85 *	0.32 ± 0.26	2.56 ± 0.35	2.89 ± 0.64	75.03 ± 10.59	0.41 ± 0.10
Often	8.9	18.96 ± 4.03	18.39 ± 6.28	0.30 ± 0.06	2.95 ± 0.92	2.64 ± 0.48	67.03 ± 30.16	0.37 ± 0.10
Fish								
Seldom	40.6	12.49 ± 1.67	8.22 ± 1.34	0.39 ± 0.05	2.43 ± 0.43	2.89 ± 0.71	58.40 ± 16.03 *	0.56 ± 0.23
Often	59.4	13.41 ± 1.42	10.63 + 1.36	0.27 ± 0.02	2.70 ± 0.47	2.85 ± 0.85	85.20 ± 12.65	0.31 ± 0.04
Furry pets at home during pregnancy								
Yes	34.7	12.35 ± 1.78	9.11 ± 1.24	0.30 ± 0.03	2.23 ± 0.41	3.09 ± 0.74	55.77 ± 11.28	0.32 ± 0.07
No	65.3	13.41 ± 1.36	9.94 ± 1.35	0.33 ± 0.03	2.78 ± 0.45	2.74 ± 0.80	84.16 ± 13.95	0.46 ± 0.14
Family smoking exposure								
Yes	18.8	8.95 ± 1.83	7.53 ± 1.22	0.35 ± 0.08	1.62 ± 0.43	2.82 ± 0.77	99.72 ± 32.60	0.32 ± 0.11
No	81.2	13.99 ± 1.24	10.14 ± 1.17	0.31 ± 0.02	2.82 ± 0.39	2.88 ± 0.69	68.44 ± 9.71	0.43 ± 0.11

Seldom: less 3 times a week; Often: more than or equal to 3 times a week; Furry pets: cats, dogs, and birds. * *p* < 0.05; ^†^ *p* < 0.01.

**Table 4:** Spearman correlation among maternal urinary phthalate metabolites, maternal total IgE, and cord blood IgE levels.

	MEP	MnBP	MBzP	MEHP	BPA	Maternal IgE	Cord Blood IgE
MEP	1.0						
MnBP	0.237 ^†^	1.0					
MBzP	0.381 ^†^	0.364 ^†^	1.0				
MEHP	0.318 ^†^	0.204 *	0.379 ^†^	1.0			
BPA	0.230 *	0.225 *	0.229 *	0.251 ^†^	1.0		
Maternal IgE	0.097	0.004	0.064	0.027	−0.064	1.0	
Cord blood IgE	0.226 *	−0.054	0.028	0.088	−0.086	0.393 ^†^	1.0

* *p* < 0.05; † *p* < 0.01.

**Table 5:** Spearman correlation among maternal urinary phthalate metabolites, maternal plasma cytokines, and cord blood plasma cytokines level.

**Maternal Plasma**							
	MEP	MnBP	MBzP	MEHP	BPA	Maternal IgE	Cord blood IgE
IL-10	0.142	0.221	−0.031	0.01	−0.122	0.091	0.089
IL-12p70	−0.171	0.072	−0.134	−0.04	0.073	0.122	−0.101
**Cord Blood**							
	MEP	MnBP	MBzP	MEHP	BPA	Maternal IgE	Cord blood IgE
IL-10	0.221 *	0.040	0.032	−0.118	−0.130	0.044	0.264 *
IL-12p70	−0.309 *	−0.036	−0.050	−0.033	−0.019	−0.083	−0.186

* *p* < 0.05.

## References

[1] Hsieh K.H., Shen J.J. (1988). Prevalence of childhood asthma in Taipei, Taiwan, and other Asian Pacific countries. J. Asthma.

[2] Asher M.I., Montefort S., Bjorksten B., Lai C.K., Strachan D.P., Weiland S.K., Williams H., Group I.P.T.S. (2006). Worldwide time trends in the prevalence of symptoms of asthma, allergic rhinoconjunctivitis, and eczema in childhood: ISAAC Phases One and Three repeat multicountry cross-sectional surveys. Lancet.

[3] Wu W.F., Wan K.S., Wang S.J., Yang W., Liu W.L. (2011). Prevalence, severity, and time trends of allergic conditions in 6-to-7-year-old schoolchildren in Taipei. J. Investig. Allergol. Clin. Immunol..

[4] Frederiksen H., Jensen T.K., Jorgensen N., Kyhl H.B., Husby S., Skakkebaek N.E., Main K.M., Juul A., Andersson A.M. (2014). Human urinary excretion of non-persistent environmental chemicals: An overview of Danish data collected between 2006 and 2012. Reproduction.

[5] Schettler T. (2006). Human exposure to phthalates via consumer products. Int. J. Androl..

[6] Kay V.R., Bloom M.S., Foster W.G. (2014). Reproductive and developmental effects of phthalate diesters in males. Crit. Rev. Toxicol..

[7] Erler C., Novak J. (2010). Bisphenol a exposure: Human risk and health policy. J. Pediatr. Nurs..

[8] Wu M.T., Wu C.F., Wu J.R., Chen B.H., Chen E.K., Chao M.C., Liu C.K., Ho C.K. (2012). The public health threat of phthalate-tainted foodstuffs in Taiwan: The policies the government implemented and the lessons we learned. Environ. Int..

[9] Vom Saal F.S., Nagel S.C., Coe B.L., Angle B.M., Taylor J.A. (2012). The estrogenic endocrine disrupting chemical bisphenol A (BPA) and obesity. Mol. Cell Endocrinol..

[10] Ku H.Y., Su P.H., Wen H.J., Sun H.L., Wang C.J., Chen H.Y., Jaakkola J.J., Wang S.L., Group T. (2015). Prenatal and postnatal exposure to phthalate esters and asthma: A 9-year follow-up study of a taiwanese birth cohort. PLoS ONE.

[11] Ait Bamai Y., Shibata E., Saito I., Araki A., Kanazawa A., Morimoto K., Nakayama K., Tanaka M., Takigawa T., Yoshimura T. (2014). Exposure to house dust phthalates in relation to asthma and allergies in both children and adults. Sci. Total Environ..

[12] Hoppin J.A., Jaramillo R., London S.J., Bertelsen R.J., Salo P.M., Sandler D.P., Zeldin D.C. (2013). Phthalate exposure and allergy in the U.S. population: Results from NHANES 2005–2006. Environ. Health Perspect..

[13] Yu H.R., Tsai C.C., Chang L.S., Huang H.C., Cheng H.H., Wang J.Y., Sheen J.M., Kuo H.C., Hsieh K.S., Huang Y.H. (2017). l-Arginine-Dependent Epigenetic Regulation of Interleukin-10, but Not Transforming Growth Factor-beta, Production by Neonatal Regulatory T Lymphocytes. Front. Immunol..

[14] Ownby D.R., McCullough J., Johnson C.C., Peterson E.L. (1996). Evaluation of IgA measurements as a method for detecting maternal blood contamination of cord blood samples. Pediatr. Allergy Immunol..

[15] Blount B.C., Milgram K.E., Silva M.J., Malek N.A., Reidy J.A., Needham L.L., Brock J.W. (2000). Quantitative detection of eight phthalate metabolites in human urine using HPLC-APCI-MS/MS. Anal. Chem..

[16] Yu H.R., Chang J.C., Chen R.F., Chuang H., Hong K.C., Wang L., Yang K.D. (2003). Different antigens trigger different Th1/Th2 reactions in neonatal mononuclear cells (MNCs) relating to T-bet/GATA-3 expression. J. Leukoc. Biol..

[17] O’Connell E.J. (2003). Pediatric allergy: A brief review of risk factors associated with developing allergic disease in childhood. Ann. Allergy Asthma Immunol..

[18] Nabavi M., Ghorbani R., Asadi A.M., Faranoush M. (2013). Factors associated with cord blood IgE levels. Asian Pac. J. Allergy Immunol..

[19] De Amici M., Perotti F., Marseglia G.L., Ierullo A.M., Bollani L., Decembrino L., Licari A., Quaglini S., Stronati M., Spinillo A. (2017). Cord and blood levels of newborn IgE: Correlation, role and influence of maternal IgE. Immunobiology.

[20] Gluckman P.D., Hanson M.A., Buklijas T. (2010). A conceptual framework for the developmental origins of health and disease. J. Dev. Orig. Health Dis..

[21] Martino D., Prescott S. (2011). Epigenetics and prenatal influences on asthma and allergic airways disease. Chest.

[22] Kimber I., Dearman R.J. (2010). An assessment of the ability of phthalates to influence immune and allergic responses. Toxicology.

[23] Gascon M., Casas M., Morales E., Valvi D., Ballesteros-Gomez A., Luque N., Rubio S., Monfort N., Ventura R., Martinez D. (2015). Prenatal exposure to bisphenol A and phthalates and childhood respiratory tract infections and allergy. J. Allergy Clin. Immunol..

[24] Whyatt R.M., Perzanowski M.S., Just A.C., Rundle A.G., Donohue K.M., Calafat A.M., Hoepner L.A., Perera F.P., Miller R.L. (2014). Asthma in inner-city children at 5–11 years of age and prenatal exposure to phthalates: The Columbia Center for Children’s Environmental Health Cohort. Environ. Health Perspect..

[25] Wang I.J., Lin C.C., Lin Y.J., Hsieh W.S., Chen P.C. (2014). Early life phthalate exposure and atopic disorders in children: A prospective birth cohort study. Environ. Int..

[26] Yu Z., Chen J., Zhang Q., Yin X., Wang Y., Fu J., Zou L., Kong W. (2015). Maternofetal transfer of antibodies and the influence of maternal atopic status on the neonate. Am. J. Rhinol. Allergy.

[27] Duty S.M., Ackerman R.M., Calafat A.M., Hauser R. (2005). Personal care product use predicts urinary concentrations of some phthalate monoesters. Environ. Health Perspect..

[28] Hoppin J.A., Brock J.W., Davis B.J., Baird D.D. (2002). Reproducibility of urinary phthalate metabolites in first morning urine samples. Environ. Health Perspect..

[29] Hauser R., Meeker J.D., Park S., Silva M.J., Calafat A.M. (2004). Temporal variability of urinary phthalate metabolite levels in men of reproductive age. Environ. Health Perspect..

[30] Ferguson K.K., McElrath T.F., Ko Y.A., Mukherjee B., Meeker J.D. (2014). Variability in urinary phthalate metabolite levels across pregnancy and sensitive windows of exposure for the risk of preterm birth. Environ. Int..

[31] Ferguson K.K., McElrath T.F., Mukherjee B., Loch-Caruso R., Meeker J.D. (2015). Associations between Maternal Biomarkers of Phthalate Exposure and Inflammation Using Repeated Measurements across Pregnancy. PLoS ONE.

[32] Shin H.M., Bennett D.H., Barkoski J., Ye X., Calafat A.M., Tancredi D., Hertz-Picciotto I. (2019). Variability of urinary concentrations of phthalate metabolites during pregnancy in first morning voids and pooled samples. Environ. Int..

[33] Sadeghnejad A., Karmaus W., Davis S., Kurukulaaratchy R.J., Matthews S., Arshad S.H. (2004). Raised cord serum immunoglobulin E increases the risk of allergic sensitisation at ages 4 and 10 and asthma at age 10. Thorax.

[34] Kjellman N.I., Croner S. (1984). Cord blood IgE determination for allergy prediction--a follow-up to seven years of age in 1651 children. Ann. Allergy.

[35] Ait Bamai Y., Miyashita C., Araki A., Nakajima T., Sasaki S., Kishi R. (2018). Effects of prenatal di(2-ethylhexyl) phthalate exposure on childhood allergies and infectious diseases: The Hokkaido Study on Environment and Children’s Health. Sci. Total Environ..

[36] Ashley-Martin J., Dodds L., Levy A.R., Platt R.W., Marshall J.S., Arbuckle T.E. (2015). Prenatal exposure to phthalates, bisphenol A and perfluoroalkyl substances and cord blood levels of IgE, TSLP and IL-33. Environ. Res..

[37] Poulsen L.K., Hummelshoj L. (2007). Triggers of IgE class switching and allergy development. Ann. Med..

[38] Ozdemir C., Akdis M., Akdis C.A. (2010). T-cell response to allergens. Chem. Immunol. Allergy.

[39] Bornehag C.G., Nanberg E. (2010). Phthalate exposure and asthma in children. Int. J. Androl..

[40] Hansen J.F., Nielsen C.H., Brorson M.M., Frederiksen H., Hartoft-Nielsen M.L., Rasmussen A.K., Bendtzen K., Feldt-Rasmussen U. (2015). Influence of phthalates on in vitro innate and adaptive immune responses. PLoS ONE.

[41] Hsieh C.S., Macatonia S.E., Tripp C.S., Wolf S.F., O’Garra A., Murphy K.M. (1993). Development of TH1 CD4+ T cells through IL-12 produced by Listeria-induced macrophages. Science.

[42] Zheng H., Ban Y., Wei F., Ma X. (2016). Regulation of Interleukin-12 Production in Antigen-Presenting Cells. Adv. Exp. Med. Biol..

[43] Kuo F.C., Su S.W., Wu C.F., Huang M.C., Shiea J., Chen B.H., Chen Y.L., Wu M.T. (2015). Relationship of urinary phthalate metabolites with serum thyroid hormones in pregnant women and their newborns: A prospective birth cohort in Taiwan. PLoS ONE.

[44] Singh S., Li S.S. (2011). Phthalates: Toxicogenomics and inferred human diseases. Genomics.

[45] Schecter A., Lorber M., Guo Y., Wu Q., Yun S.H., Kannan K., Hommel M., Imran N., Hynan L.S., Cheng D. (2013). Phthalate concentrations and dietary exposure from food purchased in New York State. Environ. Health Perspect..

[46] Chen C.C., Wang Y.H., Wang S.L., Huang P.C., Chuang S.C., Chen M.H., Chen B.H., Sun C.W., Fu H.C., Lee C.C. (2017). Exposure sources and their relative contributions to urinary phthalate metabolites among children in Taiwan. Int. J. Hyg. Environ. Health.

[47] Shen Q., Shi H., Zhang Y., Cao Y. (2015). Dietary intake and phthalates body burden in boys and girls. Arch. Public Health.

[48] Cheng Z., Nie X.P., Wang H.S., Wong M.H. (2013). Risk assessments of human exposure to bioaccessible phthalate esters through market fish consumption. Environ. Int..

